# Cellular FXIII in Human Macrophage-Derived Foam Cells

**DOI:** 10.3390/ijms24054802

**Published:** 2023-03-02

**Authors:** Laura Somodi, Emőke Horváth, Helga Bárdos, Barbara Baráth, Dávid Pethő, Éva Katona, József Balla, Nicola J. Mutch, László Muszbek

**Affiliations:** 1 Division of Clinical Laboratory Science, Department of Laboratory Medicine, Faculty of Medicine, University of Debrecen, 98 Nagyerdei krt, 4032 Debrecen, Hungary; 2 Kálmán Laki Doctoral School of Biomedical and Clinical Sciences, University of Debrecen, 98 Nagyerdei krt, 4032 Debrecen, Hungary; 3 Pathology Service, County Emergency Clinical Hospital of Targu Mures, 50 Gheorghe Marinescu Street, 540136 Targu Mures, Romania; 4 Department of Pathology, Faculty of Medicine, George Emil Palade University of Medicine, Pharmacy, Science, and Technology of Targu Mures, 38 Gheorghe Marinescu Street, 540142 Targu Mures, Romania; 5 Department of Public Health and Epidemiology, Faculty of Medicine, University of Debrecen, 26 Kassai út, 4028 Debrecen, Hungary; 6 Division of Nephrology, Department of Medicine, Faculty of Medicine, University of Debrecen, 98 Nagyerdei krt, 4032 Debrecen, Hungary; 7 ELKH-UD Vascular Pathophysiology Research Group 11003, University of Debrecen, 4032 Debrecen, Hungary; 8 Aberdeen Cardiovascular and Diabetes Centre, Institute of Medical Sciences, School of Medicine, Medical Sciences and Nutrition, University of Aberdeen, Aberdeen AB25 2ZD, UK

**Keywords:** factor XIII, foam cells, macrophages, vascular smooth muscle cells, oxidized LDL, enzyme-modified LDL, transglutaminase, atherosclerotic plaque, cross-linking

## Abstract

Macrophages express the A subunit of coagulation factor XIII (FXIII-A), a transglutaminase which cross-links proteins through Nε-(γ-L-glutamyl)-L-lysyl iso-peptide bonds. Macrophages are major cellular constituents of the atherosclerotic plaque; they may stabilize the plaque by cross-linking structural proteins and they may become transformed into foam cells by accumulating oxidized LDL (oxLDL). The combination of oxLDL staining by Oil Red O and immunofluorescent staining for FXIII-A demonstrated that FXIII-A is retained during the transformation of cultured human macrophages into foam cells. ELISA and Western blotting techniques revealed that the transformation of macrophages into foam cells elevated the intracellular FXIII-A content. This phenomenon seems specific for macrophage-derived foam cells; the transformation of vascular smooth muscle cells into foam cells fails to induce a similar effect. FXIII-A containing macrophages are abundant in the atherosclerotic plaque and FXIII-A is also present in the extracellular compartment. The protein cross-linking activity of FXIII-A in the plaque was demonstrated using an antibody labeling the iso-peptide bonds. Cells showing combined staining for FXIII-A and oxLDL in tissue sections demonstrated that FXIII-A-containing macrophages within the atherosclerotic plaque are also transformed into foam cells. Such cells may contribute to the formation of lipid core and the plaque structurization.

## 1. Introduction

Coagulation factor XIII (FXIII) present in the plasma (pFXIII) is a tetrameric pro-transglutaminase that consists of two potentially active A subunits (FXIII-A) and two inhibitory/protective B subunits (FXIII-B). The dimer of FXIII-A is also expressed in several cell types (cFXIII; FXIII-A_2_), including platelets, megakaryocytes, monocytes, macrophages, dendritic cells, chondrocytes, osteoblasts, preadipocytes, and corneal keratocytes. The transformation of pFXIII into an active transglutaminase (FXIIIa) needs proteolytic removal of an activation peptide from the N-terminus of FXIII-A and Ca^2+^ induced dissociation of FXIII-B from the tetramer. For the cellular form, the elevation of intracellular Ca^2+^ concentration is sufficient to bring about the active configuration. The function of FXIII in the plasma was elucidated a long time ago. In addition to mechanically stabilizing fibrin and protecting it from fibrinolytic degradation, it is essential for maintaining pregnancy, is important for proper wound healing, and is involved in angiogenesis. Earlier and more recent data on the structure and functions of FXIII are reviewed in references [1,2,3,4,5,6].

In the eighties we reported for the first time that monocytes and peritoneal macrophages express FXIII-A but not FXIII-B [7,8]. This finding was soon confirmed in McDonagh’s laboratory [9]. FXIIIa is a member of the transglutaminase family, other members (transglutaminase 1–7) are involved in a number of transglutaminases-related functions; an excellent review on transglutaminases in monocytes and macrophages is provided in reference [10]. The transglutaminase activity in monocytes from FXIII-A-deficient patients was below the limit of detection and only traces of tissue transglutaminase (TGM2) could be detected in freshly prepared, non-stimulated monocytes [11,12]. However, the TGM2 content rapidly increased during culturing or stimulation of the cells [12]. Monocyte/macrophage cFXIII might exert both intracellular, and if becomes externalized, extracellular function. Intracellular FXIIIa activity supports phagocytosis mediated by the Fc region of IgG and complement receptor [13]. cFXIII, lacking signal peptide, is not secreted by the usual secretory pathway, however, it could become externalized through alternative mechanisms. An unorthodox secretory mechanism is suggested by the finding that in monocytes and macrophages, in association with Golgi vesicles, FXIIIa is directed to the plasma membrane [14]. Most recently it was shown that FXIII-A becomes externalized and accumulated on the membrane of human monocytes in response to stimulation by IL-4 or IL-10 and becomes capable of exerting extracellular functions [15].

Macrophages, by uptaking lipids, particularly oxidized LDL (oxLDL), are easily transformed into foam cells. Foam cells play a vital role in the initiation of atherosclerosis and in the development of atherosclerotic plaque. In the present study, we investigated how the transformation of macrophages into foam cells influences the cFXIII content of such cells. A further question was if foam cells developed from other cells, particularly from vascular smooth muscle cells (VMSCs), also express cFXIII. We also explored the distribution of FXIII-A in the atherosclerotic plaque and attempted to verify the presence of complex protein structures cross-linked by FXIIIa.

## 2. Results

### 2.1. Investigation of Macrophage and HAoSMC-Derived Foam Cells by Immunofluorescence Microscopy for FXIII-A Expression and LDL Ingestion

**Figure 1:**
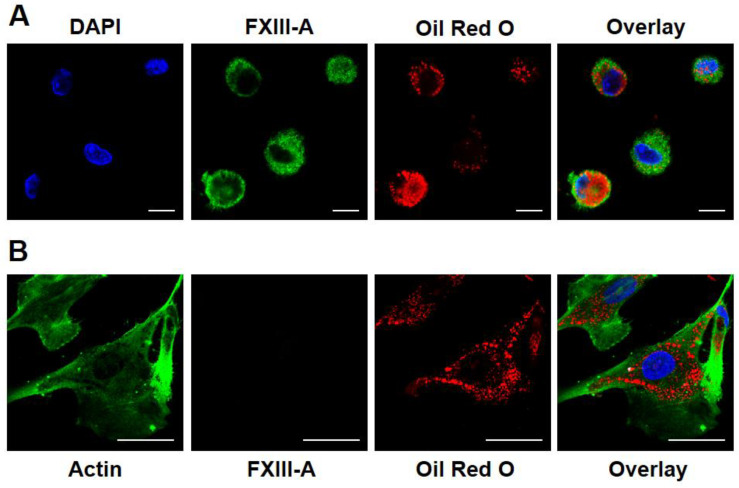
Investigation of FXIII-A expression in foam cells of different origin. (**A**) FXIII-A in macrophages transformed into foam cells by oxidized LDL ingestion. FXIII-A appears in green, while oxidized LDL is shown in red color. Scale bars correspond to 10 μm. (**B**) The lack of FXIII-A in vascular smooth muscle cells transformed into foam cells by ingestion of enzyme-modified non-oxidized LDL. Smooth muscle actin is depicted in green, while modified LDL is represented by red color. Scale bars correspond to 50 μm.

First, we investigated if FXIII-A is retained in macrophages undergoing transition into foam cells by immunohistochemistry. Human monocytes differentiated into macrophages were cultured and transformed into foam cells by ingesting oxLDL (further details are provided in Section 4.1). In Figure 1A, four macrophages, all intensively stained for FXIII-A, are shown. These macrophages accumulated oxLDL to different extents. In cells with considerable accumulation of lipid particles (two macrophages on the left side of the picture), FXIII-A became marginalized in the cytoplasm. Evidently the ingested lipid particles occupied a significant part of the central cytoplasm, pushing other cytoplasmic constituents toward the sub-membranous region.

In addition to macrophages, several other cell types present in the atherosclerotic plaque might potentially accumulate lipids and could be transformed into foam cells. Vascular smooth muscle cells, other major constituents of the atherosclerotic plaque, are also capable of transformation into foam cells. oxLDL is a relatively poor inducer of such transformation, but these cells can easily uptake enzyme-modified LDL (eLDL) that had been digested by trypsin plus cholesterol esterase. Human aortic smooth muscle cells (American Type Culture Collection) were cultured and used for investigating the ingestion of eLDL. As demonstrated in Figure 1B, these cells showed intense staining for actin, however, their transformation into foam cells was not accompanied by the expression of FXIII-A.

### 2.2. Transformation of Macrophages into Foam Cells Results in Elevated Expression of Cellular FXIII

**Figure 2:**
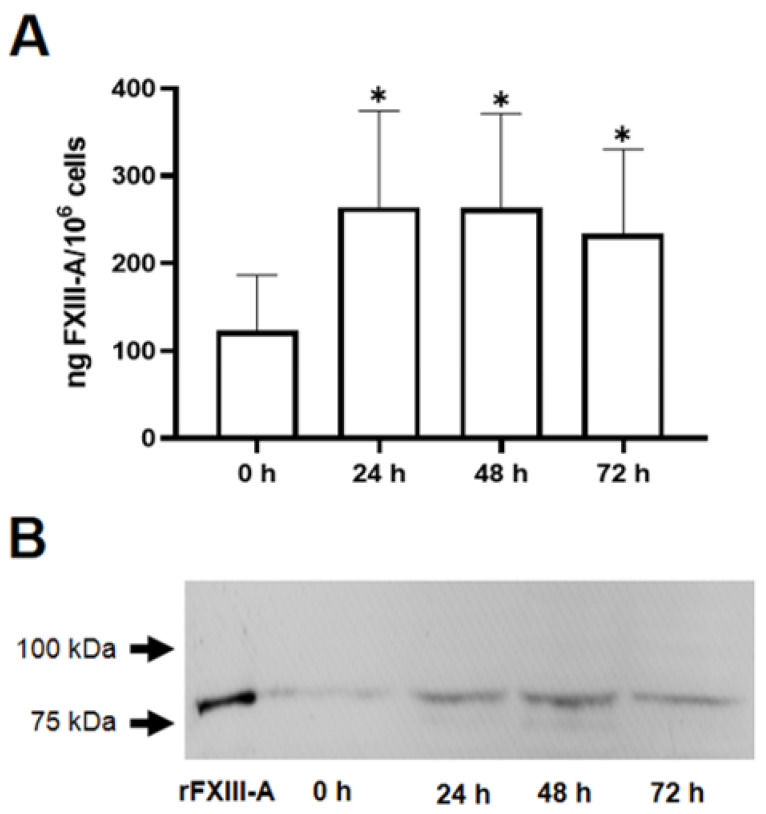
Elevation of FXIII-A in macrophages following their transformation into foam cells by a single dose of 50 μg/mL oxidized LDL (oxLDL). (**A**) FXIII-A was measured by ELISA in the lysates of non-treated cells and in cells harvested 24, 48, and 72 h after the addition of oxLDL. Statistical significance was calculated by comparing the results of oxLDL-treated cells to those of non-treated cells (*n* = 5, * *p* < 0.05). (**B**) A representative Western blot demonstrates an elevated FXIII-A level in macrophages transformed into foam cells. The arrows indicate the migration of 75 kDa and 100 kDa components of the molecular weight markers in the Precision Plus Protein Standards.

The next question we addressed was if the ingestion of oxLDL particles influences the FXIII-A content of the macrophages. Using FXIII-A ELISA, it was shown that 24 h after a single dose of oxLDL, the formed foam cells exhibited more than double FXIII-A as compared to their non-transformed counterparts (Figure 2A). The elevated FXIII-A content only slightly decreased during the following 48 h. These results were confirmed by Western blotting technique, as well. The Western blot shown in Figure 2B also demonstrates that FXIII-A content of macrophages considerably increased 24 h after the ingestion of oxLDL and only slightly decreased afterwards.

### 2.3. Macrophages and FXIII-A in the Atherosclerotic Plaque

**Figure 3:**
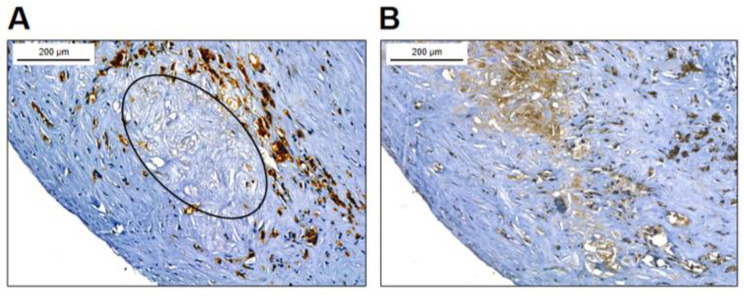
Macrophages in carotid artery plaque. (**A**) Type IV carotid artery plaque, with clearly visible lipid core, most of which is within the elliptical border. The lipid core is surrounded by CD68-positive macrophages. (**B**) FXIII-A-positive cells are detected in the plaque by immune-peroxidase staining; FXIII-A is also present extracellularly in the lipid core. More details are shown in Figure 4.

**Figure 4:**
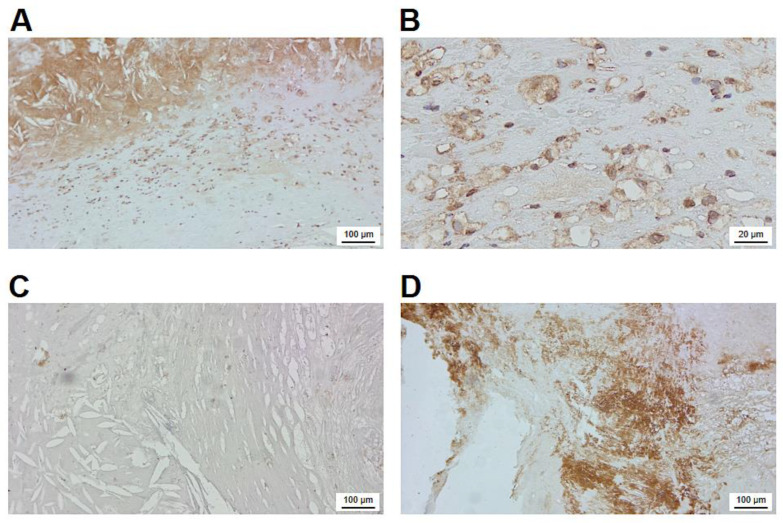
Immunohistochemical visualization of FXIII-A and cross-linked protein structures in the atherosclerotic plaque. (**A**) FXIII-A of cellular and extracellular localization in the atherosclerotic plaque. (**B**) FXIII-A-containing macrophages in the plaque at higher magnification. (**C**) Negative control for FXIII-A staining. (**D**) Protein mass cross-linked through Nε-(γ-L-glutamyl)-L-lysyl bonds in a non-cellular part of the sclerotic plaque.

After demonstrating that macrophage-derived foam cells contain a considerable amount of cellular FXIII, we explored if FXIII-A is present in the atherosclerotic plaque, and if yes, it is of intracytoplasmic and/or extracellular localization. Figure 3A demonstrates a type IV carotid artery plaque with a lipid core and surrounding CD68-positive macrophages. Another plaque stained for FXIII-A is shown in Figure 3B. Here, beside cells expressing FXIII-A staining of extracellular component can also be observed. More detailed localization of FXIII-A in the plaque is demonstrated in Figure 4.

### 2.4. Visualization of FXIII-A and Isopeptide Cross-Links within the Atherosclerotic Plaque by Immunohistochemistry

Figure 4 provides a detailed overview on the immunohistochemical localization of FXIII-A in the atherosclerotic plaque. The intense intra-cytoplasmatic brown immune-peroxidase staining demonstrates that FXIII-A is present in numerous macrophage-like cells underneath the lipid core (Figure 4A and Supplementary Figure S1A–D). In many of these cells, the empty non-stained part of the cytoplasm (Figure 4B and Supplementary Figure S1C,D) indicates that lipids that had been ingested by the cells were solubilized and removed by solvents used for the fixation/staining procedure. The intense extracellular staining of the lipid containing core of FXIII-A suggests that FXIII-A derived from the plasma and/or released from apoptotic/necrotic macrophages are bound to core constituents. The possibility of non-specific binding of primary or secondary antibodies used for the visualization of FXIII-A was excluded by the lack of staining in experiments with negative controls (Figure 4C).

A further question was if FXIII present in the atherosclerotic plaque was active and, as an active transglutaminase, if it was involved in cross-linking proteins. Using a specific antibody that detects ε-(γ-glutamyl)lysyl bonds, it was shown that the non-cellular part of the plaque is loaded by cross-linked protein structures (Figure 4D). This result clearly indicates that FXIII is not just present in the atherosclerotic plaque, but it actively contributes to its structurization.

### 2.5. FXIII-A-Containing Foam Cells within the Atherosclerotic Plaque

**Figure 5:**
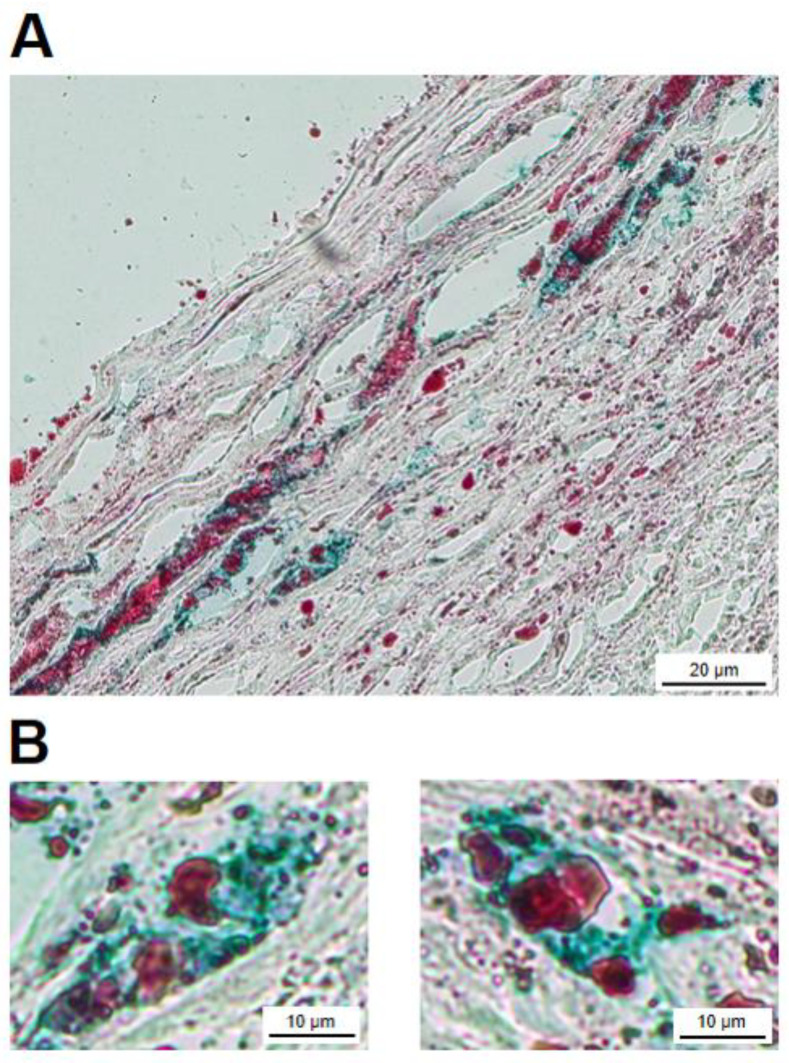
Atheromatous plaque cryosections with combined Oil Red O (ORO) and FXIII-A staining (PolyDetector HRP Green chromogen). (**A**) Numerous macrophages, some with foamy cytoplasm, in which the presence of ORO-positive vacuoles can be observed. ORO-positive droplets are also present in the extracellular compartment. (**B**) Examples of two FXIII-A-positive macrophages which were also stained with ORO.

After establishing the presence of FXIII-A-containing cells in the atherosclerotic plaque and its cross-linked protein products, it was attempted to show that part of the FXIII-A-containing macrophages housing the plaque were transformed into foam cells. In cryosections Oil Red O (ORO) stained droplets, both in the intracellular and extracellular compartments are observed (Figure 5A). Several cells show co-staining for ORO and an anti-FXIII-A antibody. Cells depicted at higher magnification clearly demonstrate such a combination (Figure 5B). This finding shows that FXIII-A-containing foam cells are not only in vitro experimental products, but also exist in vivo in the atherosclerotic plaque.

## 3. Discussion

Macrophages are multipotent, multifunctional cells which may undergo considerable transformation in response to various inducers. In different environmental conditions, they may remain non-polarized, or, by different polarizing agents, they could be transformed into cells with pro-inflammatory M1 or anti-inflammatory M2 phenotypes [16]. Recent studies distinguished three main clusters of macrophages [17,18]. The resident-like macrophages, with a phenotype resembling the M2 subtype can infiltrate the plaque. They are affected by a number of factors in the vessel wall which may influence their actual dynamic state of polarization [19]. Although the presence of FXIII in human atherosclerotic lesions has been described in 1998 by Romanic et al. [20], only a few studies have been reported on this subject. FXIII expressed in alternatively activated macrophages have been detected within the aortic valve in patients with aortic stenosis and it has been suggested to be involved in the stenosis valve progression [21].

Macrophages are capable of uptaking lipids and transforming into foam cells. Foam cells play a major role in the initiation and progression of atherosclerosis. Going through apoptosis, autophagy, necroptosis, and pyroptosis, they provide the major source of the necrotic core in the atherosclerotic plaque. In addition to macrophages, in certain conditions, vascular smooth muscle cells (VSMCs), stem/progenitor cells, and endothelial cells might also ingest lipids and become transformed into foam cells [22,23]. VSMCs-derived foam cells undergoing phenotypic transformation represent a considerable proportion, approximately 40%, of this cell type in the plaque [24]. In response to the modifications of the local environment, VSMCs switch from a contractile to a secretory phenotype and may also display macrophagic marker expression and a macrophagic behavior [25]. Expressing macrophagic markers and displaying macrophagic behavior, in theory, could involve the synthesis of FXIII-A. As shown in Figure 1, both macrophage- and VSMC-derived cells are capable of ingesting lipid particles. As the capacity of VSMCs for ingesting oxLDL is low, in these experiments we used eLDL, which was easily taken up by HAoSMCs. These cells do not express FXIII-A and its transformation into foam cells did not change the situation. This finding suggests that transformation into foam cells is not responsible for the additional FXIII-A acquired by macrophage-derived foam cells; it is not the characteristic of foam cell formation in general.

FXIII-A content of macrophages shows a drastic increase when stimulated by interleukin-4, while interferon γ fails to elicit such a change [26,27], i.e., FXIII-A content of the cells is drastically different in polarized M1 and M2 phenotypes. As the uptake of oxLDL also induces macrophage differentiation and activation toward M2 phenotype [28], we were interested in how the cellular FXIII-A content is influenced by the transformation of macrophages into foam cells. In our experiments, the FXIII-A level of the non-polarized macrophages became more than double following the ingestion of oxLDL particles (Figure 2). Although longer and more robust stimulation by IL-4 induced a considerably higher increase of cell-associated FXIII-A [26], it would be interesting to study how polarizing agents would influence the FXIII-A level in foam cells.

In tissue sections, a rather abundant cell population was stained for FXIII-A (Figure 3). Most of the FXIII-A+ cells appear as macrophage-derived foam cells; the empty part of the cells suggests the solubilization of lipid particles during fixation/staining (Figure 4). Indeed, the combination of staining for FXIII-A with the lipid stain ORO clearly demonstrated that FXIII-A and lipid droplets could be found within the same cell population (Figure 5). In the cryosections, a considerable non-cellular area of the atherosclerotic plaque also showed intensive staining for FXIII-A (Figure 4A). FXIII-A in the sclerotic core is very likely derived from foam cells that lost their integrity and participate in building up the necrotic core. A further question was if FXIII present in the atherosclerotic plaque is in an active form, i.e., is it functional and does it cross-link substrate proteins. The finding that FXIII is upregulated on the surface of human monocytes in response to stimulation by IL-4 and IL-10 suggests that it might be present in a surface-associated form as an active transglutaminase in the plaque [15]. It is also very likely that cFXIII becomes released from disintegrating cFXIII-containing cells in an active form. The main extracellular function of FXIII in tissues is the cross-linking of substrate proteins through iso-peptide bonds. Using an antibody that specifically detects iso-peptide bonds, we were able to detect cross-linked protein structures in the atherosclerotic plaque that show that FXIIIa exerts its transglutaminase activity in the extracellular compartment (Figure 4D).

In summary, the major novel findings described in the manuscript are the following:

1. The transformation of macrophages into foam cells increases their intracellular FXIII-A content, while similar transformation of VSMCs fails to produce intracellular FXIII-A.

2. FXIII-A is abundant in the atherosclerotic plaque; it is present both in plaque macrophages and in the extracellular compartment. FXIII-A-containing macrophages can also be transformed into foam cells in the atherosclerotic plaque.

3. The presence of protein structures cross-linked through iso-peptide bonds, the product of FXIII-A, suggests that at least part of FXIII-A is functioning in the plaque as an active transglutaminase. 

These findings are important for the mechanism of lipid core formation and for the structurization of the plaque.

## 4. Materials and Methods

### 4.1. Culturing of Macrophages and Induction of Foam Cell Formation

Human buffy coat from healthy donors was obtained from the Hungarian National Blood Transfusion Service. Its use for macrophage and macrophage-derived foam cell preparation was approved by the Ethics Review Board of the University of Debrecen, Faculty of Medicine in accordance with the Helsinki Declaration. It was diluted with an equal volume of sterile phosphate-buffered saline containing 5 mM EDTA (PBS-EDTA). Thirty milliliters of the cell suspension was layered onto 15 mL of Histopaque-1077^®^ (Sigma-Aldrich, St. Louis, MO, USA) and centrifuged at 400× *g* for 30 min at 25 °C. Peripheral blood mononuclear cells (PBMCs) were then aspirated and washed twice with PBS-EDTA. Monocytes were isolated by negative selection-based magnetic cell sorting (Miltenyi Biotech GmbH, Bergisch Gladbach, Germany) according to the manufacturer’s protocol. To differentiate into macrophages, monocytes were cultured in RPMI 1640 medium containing 2 mM L-glutamine and 25 mM HEPES (Life Technologies, Waltham, MA, USA), 10 µg/mL gentamycin (Krka, d. d., Novo mesto, Novo mesto, Slovenia), 10% fetal bovine serum (FBS, Life Technologies, Grand Island, NY, USA), and 50 ng/mL granulocyte-macrophage colony-stimulating factor (GM-CSF, Life Technologies, Carlsbad, CA, USA) for 3 days at 37 °C in 5% CO_2_ humidified air. Foam cells were then generated from macrophages under the same conditions by incubation with 50 µg/mL oxLDL (Life Technologies, Eugene, OR, USA) for 3 days. Cells were cultured at concentration of 10^6^ cells/mL in Teflon dishes to keep cells in suspension for use in the detection/measurement of cFXIII by enzyme-linked immunosorbent assay (ELISA) and Western blotting techniques.

### 4.2. Immunofluorescent Analysis of Macrophage-Derived Foam Cells

For immunofluorescent studies, cells were seeded onto non-treated, 8-well Lab-Tek™ chamber slides (Nunc™, Thermo Fisher Scientific, Rochester, NY, USA) at 5 × 10^5^ cells/well density. RPMI 1640 medium was removed and cells were washed three times with PBS. Adherent cells were fixed in 3.7% paraformaldehyde for 30 min, followed by rinsing with PBS. Non-specific IgG binding was blocked by normal human serum (EMD Millipore Corporation, Burlington, MA, USA) for 15 min. Foam cells were stained with rabbit anti-human FXIII-A antibody [29] for 60 min and DyLight 488-labeled goat anti-rabbit antibody (Vector Laboratories, Burlingame, CA, USA) was used as a secondary antibody for 45 min. Then, slides were washed and staining with ORO (Sigma-Aldrich) was performed for 15 min, followed by washing with distilled water three times. To counterstain nuclei, Vectashield^®^ mounting medium with DAPI (Vector Laboratories) was used. Staining steps were carried out at room temperature in the dark. Slides were investigated with Zeiss LSM 700 confocal microscope (Zeiss, Oberkochen, Germany) and solid-state diode lasers (405 nm, 488 nm, and 555 nm). Detection of the fluorescence signals was performed by selective laser excitation coupled to efficient splitting of the emitted light using a variable secondary dichroic (VSD) beam-splitter.

### 4.3. Preparation of Enzyme-Modified LDL (eLDL)

A slight modification of the method described by Bhakdi et al. [30] was used for the generation of eLDL. Briefly, human native LDL (density 4 mg/mL) from plasma of healthy donors was isolated by ultracentrifugation [31]. LDL was diluted to 2 mg/mL in 20 mM HEPES, 150 mM NaCl, 2 mM CaCl_2_, pH 7.0. For enzymatic modification, LDL was digested with 4 µg/mL trypsin from bovine pancreas (Sigma-Aldrich) at 37 °C for 6 h and with 24 µg/mL cholesterol esterase from Pseudomonas sp. (Sigma-Aldrich) for an additional 6 h at 37 °C. Then, another 4 µg/mL trypsin and 36 µg/mL cholesterol esterase were added and the mixture was incubated at 37 °C for 24 h. Finally, trypsin activity was blocked by 10 µg/mL soybean trypsin inhibitor (Roche Diagnostics, Mannheim, Germany) for 60 min at 37 °C and modified LDL was dialyzed against PBS.

### 4.4. Human Aortic Smooth Muscle Cell (HAoSMC)-Derived Foam Cell Formation

HAoSMCs were purchased from American Type Culture Collection (ATCC; Manassas, VA, USA). Naive cells (donor: 38 years old healthy Caucasian male) were maintained in Dulbecco’s Modified Eagle Medium (DMEM, Gibco™, Thermo Fisher Scientific) containing 1 mM sodium pyruvate, 2 mM L-glutamine, 10% FBS, and 1% gentamycin in a T75 flask at 37 °C in 5% CO_2_ humidified air. Cells were grown to 90% confluence and used at passage 8, then seeded onto non-treated 4-well chamber slides (Nunc™, Thermo Fisher Scientific) under the same conditions for 24 h. At the end of the incubation, adherent HAoSMCs were washed three times with PBS and treated with 75 µg/mL eLDL or with native LDL in DMEM medium for further 24 h.

### 4.5. Immunofluorescent Analysis of HAoSMCs-Derived Foam Cells

After a 24-h treatment by eLDL, DMEM medium was removed and slides were washed three times with PBS. Cells were fixed by a 58:2 mixture of methanol and acetic acid for 15 min. Cells were washed again and then blocking with normal human serum was performed for 15 min. HAoSMCs were stained by rabbit anti-human FXIII-A antibody (1:200 dilution) or mouse anti-human alpha-smooth muscle actin antibody (1:250 dilution, Thermo Fisher Scientific) for 60 min. After washing with PBS, DyLight 488-labeled goat anti-rabbit or DyLight 488-labeled goat anti-mouse secondary antibodies were added for 45 min. To visualize eLDL uptake by HAoSMCs, ORO staining was carried out for 15 min, then cells were washed with distillated water. Finally, slides were mounted by Vectashield^®^ mounting medium with DAPI and investigated using Zeiss LSM 700 confocal microscope.

### 4.6. Quantification of FXIII-A by ELISA in Macrophage-Derived Foam Cells

For the detection of FXIII-A, cells from macrophages and macrophage-derived foam cells cultures were removed every day and centrifuged at 200× *g* for 10 min at 25 °C. Pellets were resuspended in the mixture of PBS, 1% Triton, 120 µg/mL 2-methyl-4-isothiazolin-3-one (MIT, Sigma-Aldrich), and 1x SIGMAFAST™ Protease inhibitor (Sigma-Aldrich). Samples were stored at +4 °C until measurement. FXIII-A antigen concentration was determined by sandwich ELISA [32]. Results were adjusted to 10^6^ cells. Statistical analysis was carried out by GraphPad Prism 8.01. Distribution of the results was investigated by the Kolmogorov–Smirnov test and paired *t*-test was used for calculating the level of significance. *p* < 0.05 was considered statistically significant. Means represent the results of 5 measurements.

### 4.7. Western Blotting

Cultured cells were collected daily and centrifuged at 200× *g* for 10 min at 25 °C. Cell pellets were resuspended in an SDS-PAGE sample buffer containing 8 M urea, then denatured in boiling water for 5 min. After reduction, by adding 5% 2-mercaptoethanol proteins of equal number, lysed cells were separated by SDS-PAGE (7.5% gel), followed by transfer to PVDF membrane. Affinity purified sheep anti-FXIII-A antibody (1:3000 dilution, Affinity Biologicals, Ancaster, Hamilton, ON, Canada), biotinylated anti-sheep IgG (1:1000 dilution, Vector Laboratories), avidin-biotinylated peroxidase complex (Vector Laboratories), and ECL chemiluminescent reagent (ECL Plus+, Amersham, Little Chalfont, UK) were used for the immune reaction detecting FXIII-A in the cell lysates. Results were compared to that obtained with 100 ng of recombinant FXIII-A (Novo Nordisk A/S, Bagsvaerd, Denmark). Precision Plus Protein Standards, Dual Color (Bio-Rad, Hercules, CA, USA) was used as a molecular weight marker to indicate the molecular weight close to the migration of FXIII-A.

### 4.8. Investigation of Atherosclerotic Plaque by Immunohistochemistry

Tissue fragments harvested by conventional transluminal angioplasty from 7 patients diagnosed with symptomatic carotid artery (CA) atherosclerosis were fixed in 4% formaldehyde and embedded in paraffin. Histological features of carotid plaques were examined in 5 µm sections stained with hematoxylin and eosin. Using the criteria of the American Heart Association [33], clinically relevant type IV plaques (known also as “atheroma”) were selected for further investigations. Macrophages were visualized by immunohistochemistry using anti-CD68 mouse monoclonal antibody, clone KP1 (Immunologic, Duiven, The Netherlands). Anti-FXIII-A rabbit polyclonal antibody was used (Thermo Fisher Scientific, Fermont, CA, USA) for the detection of cellular and extracellular localization of FXIII-A. In parallel experiments, Nε-(γ-L-glutamyl)-L-lysyl isopeptide bonds were detected by an antibody purchased from Covalab (Villeurbanne, France). EnVision FLEX/HRP (Agilent, Dako Santa Clara, CA, USA) was used as a secondary antibody in combination with 3,3′-diaminobenzidine chromogen (DAB) substrate to give the reaction product a brown color. Nuclei were counterstained with hematoxylin. For negative control, normal serum was substituted for the primary antibody.

### 4.9. Lipids in FXIII-A-Positive Cells of the Atherosclerotic Plaque

Neutral lipids were visualized in cryosections according to the protocol for ORO staining solution. Anti-FXIII-A primary antibody, in combination with ORO, was used for demonstrating the intracellular presence of FXIII-A in foam cells. Briefly, after fixation in isopropyl alcohol and endogenous peroxidase blocking, a polyclonal antibody against FXIII-A was added and incubated overnight in a thermostat at 56 °C. After subsequent washing, secondary antibody was added (30 min), which was followed by immersion in ready to use ORO solution for 5 min. The slides were then rinsed with running tap water, and color development was carried out by PolyDetector HRP Green (Bio SB, Santa Barbara, CA, USA) following the manufacturer’s instructions. FXIII-A-positive macrophages shown in green were considered positive also for ORO when red intracytoplasmic vacuoles were present.

## Data Availability

Not applicable.

## Supplementary Materials

The following supporting information can be downloaded at: https://www.mdpi.com/article/10.3390/ijms24054802/s1.
