# Supplementary material for: Cellular FXIII in Human Macrophage-Derived Foam Cells
Source: Int J Mol Sci. 2023 Mar 2;24(5):4802. doi: 10.3390/ijms24054802 (PMC10002485; doi:10.3390/ijms24054802)
Supplement: Supplementary file 1 [file ijms-24-04802-s001.zip › ijms-2217472-Supplementary.pdf]

Supplementary Material

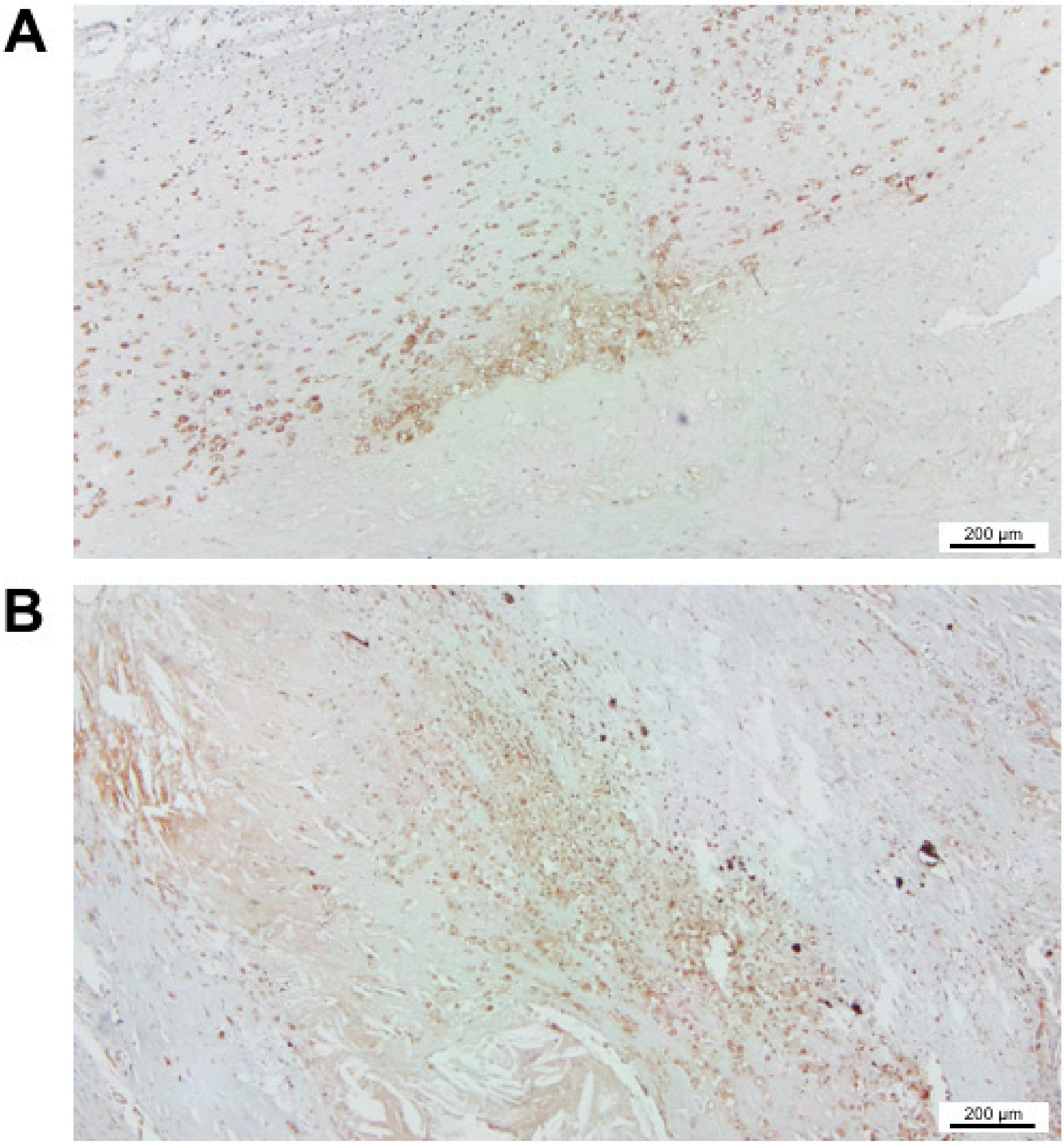

C

D

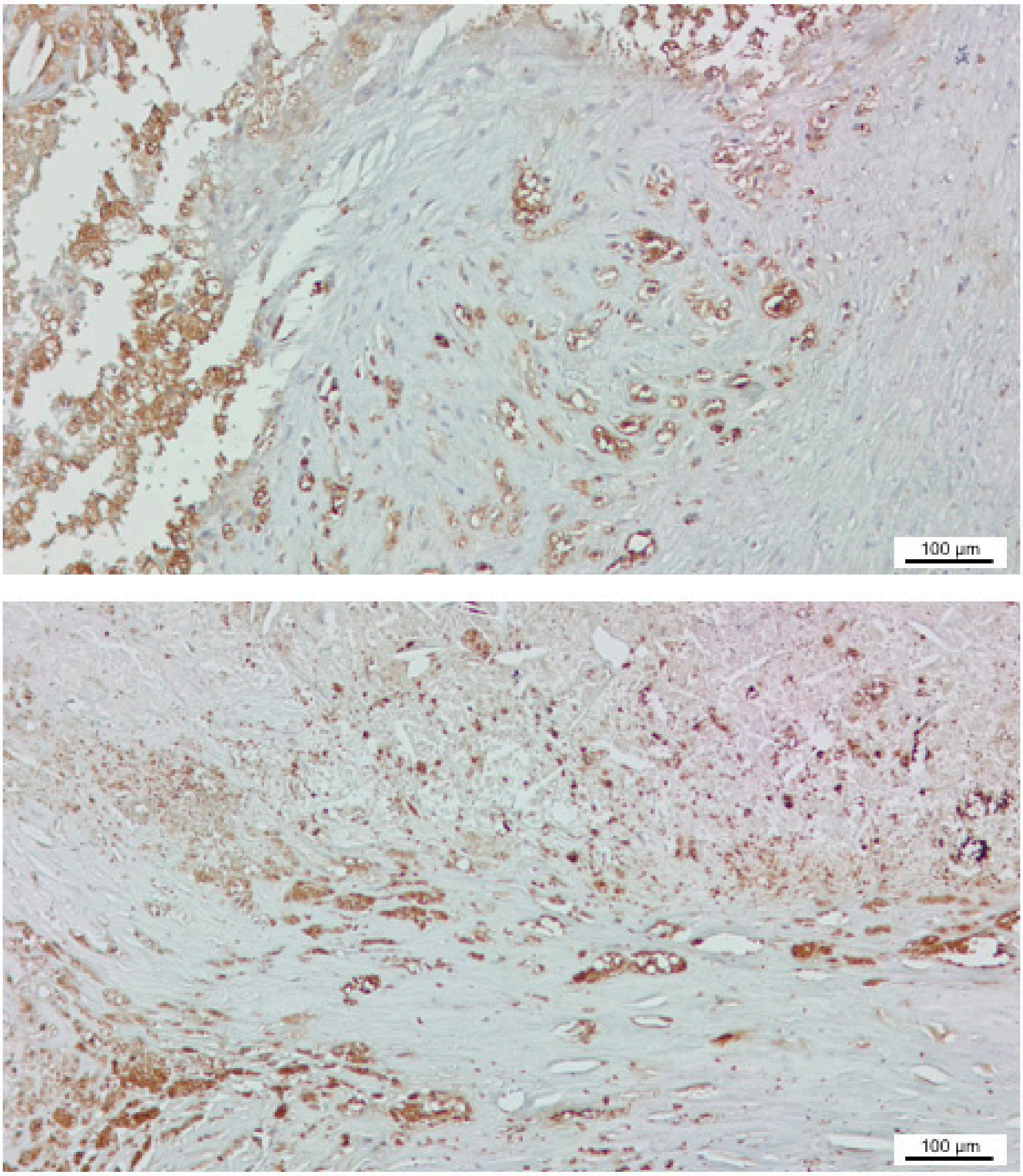

Supplementary Figure S1. Representative images from the central region of four atherosclerotic plaques developed in the carotid artery. Images from two plaques (A,B) are shown at lower and from two other ones (C,D) at higher magnification. Immunohistochemistry visualized by 3,3'-diaminobenzidine chromogen reveals both intracytoplasmic and extracellular localization of FXIII-A. Macrophages with empty, non-stained parts in the cytoplasm indicate that lipids that had been ingested by the cells were solubilized and removed by solvents used for the fixation/staining procedure. Such macrophages originally rich in lipid particles (foam cells) are extended also in the fibrotic areas and all of them are intensively stained for FXIII-A. Expression of FXIII-A is much stronger at the periphery of cells due to intracytoplasmic co-localization with lipid droplets.
